# Comparison of rheumatoid arthritis composite disease activity indices and residual activity in a Brazilian multicenter study- REAL study

**DOI:** 10.1371/journal.pone.0273789

**Published:** 2022-09-29

**Authors:** Isabela Araújo Santos, Renê Donizeti Ribeiro de Oliveira, Sergio Couto Luna Almeida, Ana Beatriz Vargas-Santos, Rodrigo Balbino Chaves Amorim, Ana Paula Monteiro Gomides, Cleandro Pires de Albuquerque, Manoel Barros Bertolo, Maria Fernanda Brandão Resende Guimarães, Maria Raquel da Costa Pinto, Gustavo Gomes Resende, Rina Dalva Neubarth Giorgi, Nathalia de Carvalho Saciloto, Sebastião Cezar Radominski, Fernanda Maria Borghi, Karina Rossi Bonfiglioli, Henrique Carrico da Silva, Maria de Fatima L. da Cunha Sauma, Ivanio Alves Pereira, Glaucio Ricardo Werner de Castro, Claiton Viegas Brenol, Ricardo Machado Xavier, Licia Maria Henrique Mota, Paulo Louzada-Junior, Geraldo da Rocha Castelar-Pinheiro

**Affiliations:** 1 Universidade de São Paulo, Ribeirão Preto, Brazil; 2 Universidade do Estado do Rio de Janeiro, Rio de Janeiro, Brazil; 3 Universidade de Brasılia, Brasılia, Brazil; 4 Universidade Estadual de Campinas, Campinas, Brazil; 5 Universidade Federal de Minas Gerais, Belo Horizonte, Brazil; 6 Hospital do Servidor Publico Estadual de São Paulo, São Paulo, Brazil; 7 Universidade Federal do Paraná, Curitiba, Brazil; 8 Universidade Estadual de Maringá, Maringá, Brazil; 9 Universidade de São Paulo, São Paulo, Brazil; 10 Universidade Federal do Para, Belém, Brazil; 11 Universidade Federal de Santa Catarina, Florianópolis, Brazil; 12 Universidade do Sul de Santa Catarina-Unisul, Florianópolis, Brazil; 13 Universidade Federal do Rio Grande do Sul, Porto Alegre, Brazil; Nippon Medical School, JAPAN

## Abstract

**Introduction:**

Rheumatoid arthritis (RA) composite disease activity indices have become handy tools in daily clinical practice and crucial in defining remission or low disease activity, the main target of the RA treatment. However, there is no definition of the best index to assess disease activity in clinical practice.

**Objectives:**

To compare the residual activity among the indices with the ACR/EULAR remission criteria (Boolean method) to identify the most feasible for assessing remission in daily practice, also considering correlation and concordance, sensibility, and specificity.

**Patients and methods:**

We selected 1116 patients with established RA from the real-life rheumatoid arthritis study database—REAL. The composite disease activity indices—DAS28-ESR, DAS28-CRP, SDAI, and CDAI–and their components were compared to the Boolean method to identify residual activity using binomial regression. The indices were analyzed for correlation and agreement using the Spearman index and weighted kappa. The chi-square test evaluated sensibility and specificity for remission based on the Boolean method.

**Results:**

DAS28-CRP overestimated remission and confirmed higher residual activity than SDAI and CDAI. The indices showed good correlation and agreement, with a better relationship between SDAI and CDAI (*k*:0,88). CDAI and SDAI showed higher sensitivity and specificity for remission based on the Boolean method. CDAI was performed in 99% of patients, while DAS28 and SDAI were completed in approximately 85%.

**Conclusions:**

Although all composite indices of activity can be used in clinical practice and showed good agreement, CDAI and SDAI have better performance in evaluating remission based on the Boolean method, showing less residual activity and higher sensibility and specificity. In addition, CDAI seems to be more feasible for disease activity evaluation in daily clinical practice, especially in developing countries.

## Introduction

Rheumatoid arthritis (RA) is a systemic inflammatory disease characterized by erosive peripheral joint involvement that can lead to structural damage, deformities, functional impairment, and poor quality of life. The worldwide prevalence is 0.2 to 1%, concordant with Brazilian data [1–4].

In the past decades, RA treatment has undergone a significant transformation. The use of disease-modifying anti-rheumatic drugs (DMARDs) under a strict treatment regimen (treat-to-target or T2T) reduced the loss of function, improved quality of life, and reduced radiographic progression compared to previous strategies [5].

The composite disease activity indices, better than individual variables, became crucial in achieving remission, the primary treatment target of RA [6–8]. The *28-joint Disease Activity Score* (DAS28) was validated in 1995 with an erythrocyte sedimentation rate (ESR) as an inflammatory marker. In 2004, C-reactive protein (CRP) was included in the formula [9, 10]. Nevertheless, they were more associated with significant residual activity disease activity. On the other hand, the *Simplified Activity Index* (SDAI) and the *Clinical Activity Index* (CDAI) became better alternatives for evaluating inflammatory activity and classification of remission with a more simplified calculation [11, 12].

In 2011, the ACR/EULAR provided Boolean and index-remission criteria for trials and clinical practice [13]. Characterized by a state of absence or minimal inflammatory activity, remission can be achieved by two methods: (1) Boolean method, defined by painful joint count (TJC) ≤1, swollen joint count (SJC) ≤1, CRP (mg/dL) ≤ 1 and global patient assessment (PtGA; 0–10 cm scale) ≤ 1; or (2) SDAI ≤ 3,3 [13]. Furthermore, remission should define a state of minimal residual disease activity with no more than two involved joints, swollen and/or tender [13].

In daily practice, all of the four indices can be used to assess inflammatory activity. Although the increased evidence of residual activity over the years, DAS28 is still the most common and well-established index in clinical practice. It also has a significant difference in weight among the variables in the formula and influences comorbidities that can contribute to inadequate remission status or low disease activity [14]. On the other hand, SDAI and CDAI show less residual activity, more accessible formulas, and greater agility and treatment definition [15–17]. SDAI shows higher agreement when compared to magnetic resonance image (MRI), and CDAI allows guiding the treatment even when laboratory tests are not available [18, 19]. Balsa et al. already proposed the superiority of SDAI over DAS28 to predict ultrasound remission in patients with RA and found that SDAI was the index closest to remission by the absence of a signal at power doppler [20]. As a result, SDAI and CDAI have been more recommended in the classification of remission, with evidence of less residual activity and higher agreement with imaging methods [20–22].

The objectives of this study were to compare the residual activity among indices based on the Boolean method of remission in a real-life setting and evaluate the most feasible for assessing remission in daily practice, also considering correlation and concordance, sensibility, and specificity.

## Material and methods

This study was part of a multicenter prospective observational cohort study—Rheumatoid Arthritis in Real Life (REAL). The objectives were to describe the demographic, clinical, and therapeutic characteristics of Brazilian patients with RA and evaluate adherence to treatment, the safety of pharmacologic therapy, and its impact on physical function and quality of life [23].

Thirteen tertiary public healthcare centers specialized in RA management were selected to represent the five geographic regions of Brazil, but only eleven from four areas were enrolled in the program. Patients were followed for 12 months with systematic data collection at the initial visit (baseline), intermediate visit (6 months ± 1 month), and final visit (12 months ± 1 month). The recruitment period started on 12 August 2015 and ended on 15 April 2016. The sample selection was by convenience. The present article is a cross-sectional evaluation of the baseline data. The study was approved by the National Commission of Ethics in Research—Ministry of Health (number 45781015810015259) and by the Research Ethics Committee of Ribeirão Preto Clinical Hospital, Ribeirão Preto Medical School of the University of São Paulo (number 45781015820015440). Each of the centers also obtained approval from the respective Institutional Review Boards. All participants signed the written consent form.

The inclusion criteria were: (1) patients with RA who fulfilled the 1987 American Rheumatism Association (ARA) or the 2010 American College of Rheumatology (ACR)/ European League Against Rheumatism (EULAR) classification criteria for rheumatoid arthritis [24, 25]; (2)18 years or older; (3) documented medical record data of at least six months of follow-up in their healthcare center before study enrollment; (4) good adherence to drug treatment; (5) adequate intellectual and communication levels to answer the questionnaires. Exclusion criteria were comorbidities that compromised the evaluation of the variables, such as stroke, chronic renal dialysis, malignant neoplasia, or major depression.

Each center enrolled about 100 patients consecutively, evaluated by the same rheumatologist. Most data were collected during medical appointments, with medical records used as secondary sources. The clinical assessment consisted of the examination of the number of tender (TJC) and swollen joints (SJC), a global health assessment (GH), a visual analog scale of disease activity by the patient (PtGA) and the physician (PhGA), and markers of inflammatory activity—erythrocyte sedimentation rate (ESR) or C-reactive protein (CRP)- to calculate the composite disease activity indices. The exams were collected with a maximum three-month interval between the visits, and qualitative values were not considered.

The patients were classified according to demographic and clinical data (time from symptom onset to diagnosis, disease duration, lifestyle habits, comorbidities, presence of erosive disease, and laboratory tests, including rheumatoid factor and anti-citrullinated protein antibody). These variables were listed in the descriptive statistical analysis. Other variables and clinical data were detailed in separate articles [23, 26–29].

The composite disease activity indices evaluated were DAS28-ESR, DAS28-CRP, SDAI, and CDAI. The indices definitions and formulas are shown in Tables 1 and 2.

**Table 1:** Composite disease activity indices and classifications [9, 11, 30, 31].

Indices	Components	Disease Activity			
		Remission	Low	Moderated	Elevated
***DAS28-ESR***	SJC (28)				
	TJC (28)	< 2,6	2,6 to 3,2	˃ 3,2 to ≤ 5,1	˃ 5,1
	ESR (mm)				
	GH (mm)				
***DAS28-CRP***	SJC(28)				
	TJC (28)	< 2,6	2,6 to 3,2	˃ 3,2 to ≤ 5,1	˃ 5,1
	CRP (mg/L)				
	GH (mm)				
***SDAI***	SJC (28) + TJC (28) +				
	CRP (mg/dL) + PtGA (cm) + PhGA (cm)	≤ 3,3	˃ 3,3 to 11	˃ 11 to ≤ 26	˃ 26
***CDAI***	SJC (28) + TJC (28) +				
	PtGA (cm) + PhGA (cm)	≤ 2,8	˃ 2,8 to 10	˃ 10 to ≤ 22	˃22
***ACR/EULAR***	**SDAI**	≤ 3,3			
	**Boolean**:	**OR**			
	SJC / TJC/ PtGA(cm)/ CRP (mg/dL)	≤1			

DAS28-CRP: Disease Activity Score 28- C reactive protein; DAS28-ESR: Disease Activity Score 28 -erythrocyte sedimentation rate; SDAI: Simplified Activity Index; CDAI: Clinical Activity Index; SJC: swollen joint count; TJC: tender joint count; GH: global health assessment (0–100 mm); PtGA: patient global assessment (0–10 cm); PhGA: physician global assessment (0–10 cm); ESR: erythrocyte sedimentation rate; CRP: C reactive protein.

**Table 2:** Composite disease activity indices formulas.

Composite Disease Activity Index Formula	
**DAS28-ESR**	0,56 x √TJC28 + 0,28 x √SJC28 + 0,70 x lnESR + 0,014 x GH
**DAS28-CRP**	0,56 x √TJC28 + 0,28 x √SJC28 + 0,36 x ln(CRP+1) + 0,014 x GH + 0,96
**SDAI**	SJC + TJC+ PtGA + PhGA + CRP (mg/dL)
**CDAI**	SJC + TJC+ PtGA + PhGA

DAS28-CRP: Disease Activity Score 28- C reactive protein; DAS28-ESR: Disease Activity Score 28 -erythrocyte sedimentation rate; SDAI: Simplified Activity Index; CDAI: Clinical Activity Index; SJC: swollen joint count; TJC: tender joint count; GH: global health assessment (0–100 mm); PtGA: patient global assessment (0–10 cm); PhGA: physician global assessment (0–10 cm; ESR: erythrocyte sedimentation rate; CRP: C reactive protein.

The epidemiological data were analyzed using descriptive statistics, and the results are presented as mean and standard deviation or median and interquartile range, as appropriate.

The indices were compared to the Boolean method using binomial regression with identity link function and random effects since the same patient could be in remission for more than one index simultaneously. Residual activity was characterized by the proportion of patients with off-target components. Although residual activity is not only characterized by activity disease scores, and symptoms like fatigue, sleep disturbances, and pain are recognized as necessary for measuring residual activity, they were not evaluated in this article.

Correlation between pairs of indices was evaluated using the Spearman index since the indices were not normally distributed. The agreement was assessed by kappa and weighted Kappa coefficient, and the indices were transformed into ordinal categoric variables, including remission, low, moderate, and elevated activity.

The indices were compared for sensibility and specificity for remission based on the Boolean method by the chi-square test. Values of p<0.05 were considered significant for all analyses. Data were analyzed using SAS software, version [9.2].

## Results

One thousand one hundred and sixteen (1116) patients were enrolled in the present cohort. Their demographics and clinical and laboratory characteristics are shown in Table 3.

**Table 3:** Baseline clinical data of patients with RA in the REAL study (n = 1116).

Clinical data	Absolute value or % (n)
**Age, years (mean ± SD)**	56.7 ± 11.4
**Female gender (%)**	998 (89.5%)
**Study time, years (mean ± SD)**	8 ± 4.2
**Ethnicity/race/color (%)**	
• **White**	634 (56.8%)
• **Black**	122 (10.9%)
• **Pardo**^a^	349 (31.2%)
**Symptom’s duration, years (mean ± SD)**	14.75 ± 7.8
**Diagnostic time interval, months (mean ± SD) [median (IQR)]**	32 ±51 12 (6–36)
**Disease duration, years (mean ± SD) [median (IQR)]**	12.6 ± 7.6 12.7 (0.7–56.9)
**Positive rheumatoid factor (%)**	78.5% (n = 1098)
**Positive anti-citrullinated peptide antibody (%)**	76.8% (n = 479)
**Erosive disease (%)**	54.9 (n = 1095)
**Fibromyalgia (%)**	154 (13.7%)
**Current smoking (%)**	121 (10.8%)
**Medications**:	
• **Synthetic DMARD**	1031 (90,9%)
• **Biological DMARD**	398 (35.7%)
• **Biological DMARD monotherapy**	62 (5.6%)
• **Glucocorticoids**	528 (47,4%)
**Pain**^b^ **(0–100) [median (IQR)]**	40 (0–100) (n = 1116)
**Fatigue (0–100) [median (IQR)]**	40 (0–100) (n = 1116)
**Global health assessment (0–100)**	38 (0–100) (n = 1116)
**ESR median (IQR)**	21 (10–40) (n = 925)
**CRP median (IQR)**	0.67 (0.25–1.78) (n = 947)
**HAQ median (IQR)**	0.875 (0.25–1.5) (n = 1112)
**DAS28-CRP median (IQR)**	3.04 (2.23–4.21) (n = 937)
**DAS28-ESR median (IQR)**	3.52 (2.55–4.51) (n = 925)
**SDAI median (IQR)**	10.59 (4.47–20.20) (n = 943)
**CDAI median (IQR)**	9 (3.7–18.89) (n = 1114)

^a^ Mixed white and black ethnicities.

^b^ Patient global assessment.

DMARDS: disease -modifying anti-rheumatic drugs; ERS: erythrocyte sedimentation rate; CRP: C- reactive protein; HAQ: health assessment questionnaire; DAS28-CRP: Disease Activity Score 28- C reactive protein; DAS28-ESR: Disease Activity Score 28 -erythrocyte sedimentation rate; SDAI: Simplified Activity Index; CDAI: Clinical Activity Index.

Approximately 90% of patients were female, with a mean age of 56.7 years and a median disease duration of 12.7 years. Most patients were white (56.8%), with a median educational time of 8 years. The seropositivity rate was almost 80% for both rheumatoid factor and ACPA. A low rate of smoking (10.8%) and fibromyalgia (13,7%) was observed. Median HAQ-DI was 0.875, with mild functional loss. DAS28-ESR median was 3.5 and DAS28-CRP 3.04, SDAI 10.5, and CDAI 9, all representing low disease activity but revealing approximately 50% of patients presenting moderate to high disease activity.

By classifying patients according to disease activity, approximately 50% had low activity or remission, except for DAS28-ESR, whose rate was around 40% (Table 4). Although 50,8% of patients (567) presented remission in at least one index, this rate decreases substantially, achieving only 12.4% (139) when considering the Boolean remission criteria. This value is similar to 10,4% (117), the rate of patients with remission in all the indices simultaneously.

**Table 4:** Classification of disease activity according to composite indices.

RA Activity	DAS28-ESR	DAS28-CRP	SDAI	CDAI
**High**	156 (16.8%)	110 (11,7%)	145 (15.4%)	214 (19.2%)
**Moderated**	387 (41.8%)	327 (34.9%)	310 (32.9%)	307 (27.6%)
**Low**	139 (15%)	147 (15.7%)	308 (32.7%)	370 (33.2%)
**Remission**	243 (26.4%)	353 (37.7%)	180 (19%)	223 (20%)
**Total**	925 (100%)	937 (100%)	943 (100%)	1114(100%)
**Losses**	191	179	173	2

DAS28-CRP: Disease Activity Score 28- C reactive protein; DAS28-ESR: Disease Activity Score 28 -erythrocyte sedimentation rate; SDAI: Simplified Activity Index; CDAI: Clinical Activity Index.

**Fig 1:**
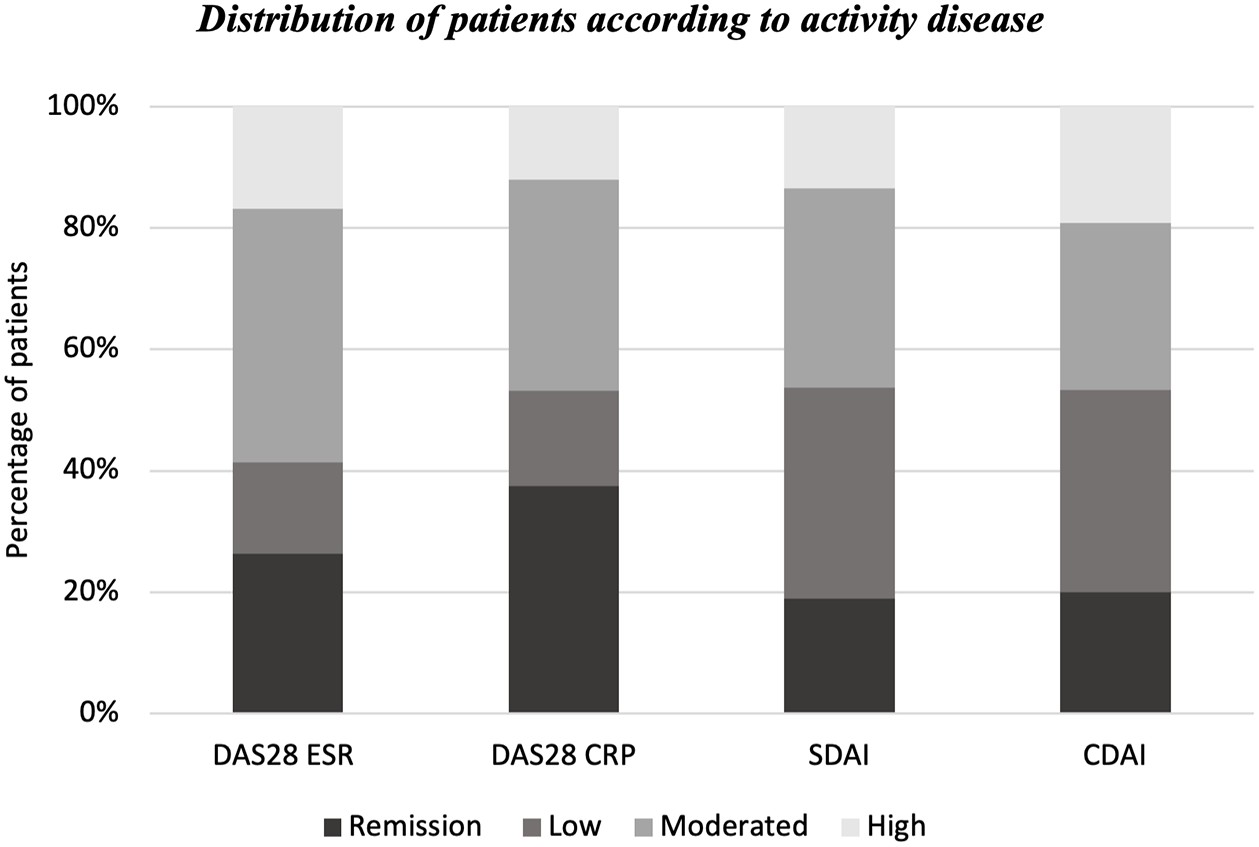
Distribution of patients according to disease activity. DAS28-CRP: Disease Activity Score 28- C reactive protein; DAS28-ESR: Disease Activity Score 28—erythrocyte sedimentation rate; SDAI: Simplified Activity Index; CDAI: Clinical Activity Index.

However, there is a difference in activity classification, and the indices disagreed when classifying low activity and remission, with a higher rate of low activity status by SDAI and CDAI than remission and a higher percentage of remission status by DAS28, both ESR and CRP (Fig 1).

The components of the indices were evaluated according to residual activity, which was characterized as the proportion of patients that did not meet the Boolean remission criteria and presented values superior to 1 in all components of the latter (Table 5). The estimated difference rates between each index were analyzed according to each component and described in Table 6.

**Table 5:** Residual activity according to various remission activity states.

Residual Activity—Boolean parameters	DAS28-ESR (n = 243)	DAS28- CRP (N = 353)	SDAI (n = 180)	CDAI (n = 223)
**TJC> 1, n (%)**	16 (6.5%)	11 (3.1%)	2 (1.1%)	1 (0.4%)
**SJC> 1, n (%)**	14 (5.7%)	34 (9.6%)	2 (1.1%)	3 (1.3%)
**CRP> 1mg/dL, n (%)**	41 (17.9%)	64 (18.1%)	26 (14.4%)	52 (26.3%)
**GH > 1 cm, n (%)**	117 (48.1%)	182 (51.5%)	61 (33.8%)	72 (32.2%)
**PtGA >1 cm, n (%)**	111 (45.6%)	165 (46.7%)	26 (14.4%)	33(14.7%)
**PhGA >1 cm, n (%)** *	71 (29.2%)	120 (33.9%)	18 (10%)	22 (10%)

DAS28-CRP: Disease Activity Score 28- C reactive protein; DAS28-ESR: Disease Activity Score 28 -erythrocyte sedimentation rate; SDAI: Simplified Activity Index; CDAI: Clinical Activity Index; TJC: tender joint count; SJC: swollen joint count; CRP: C reactive protein; GH: global health assessment; PtGA: patient’s global assessment; PhGA: physician’s global assessment.

* PhGA is not part of Boolean criteria

**Table 6:** Component estimated difference according to the composite activity indices.

Boolean parameters	Estimated difference (%)	CI (95%)	*p*
**CRP> 1**			
CDAI vs. DAS28-CRP	8.27	3.87;12.66	<0.01
CDAI vs. DAS28-ESR	8.49	2.98;14.01	<0.01
CDAI vs. SDAI	11.95	7.84-;16.06	<0.01
DAS28-CRP vs. SDAI	3,69	-0,49; 7,86	0,08
DAS28-ESR vs. SDAI	3,46	-1,89;8,81	0,21
**SJC> 1**			
CDAI vs. DAS28-CRP	-8.29	-11.42; -5.16	<0.01
CDAI vs. DAS28-ESR	-4.42	-7.48;-1.35	<0.01
CDAI vs. SDAI	0.23	-0.69; 1.16	0.62
DAS28-CRP vs. SDAI	8.52	5.45;11.59	<0.01
DAS28-ESR vs. SDAI	4.65	1.62; 7.68	<0.01
**TJC > 1**			
CDAI vs. DAS28-CRP	-2.67	-4.68; -0.66	<0.01
CDAI vs. DAS28-ESR	-6.14	-9.37; -2.91	<0.01
CDAI vs. SDAI	-0.66	-1.76;0.44	0.24
DAS28-CRP vs. SDAI	2.1	-0.35;4.36	0.10
DAS28-ESR vs. SDAI	5.47	2.02;8.92	<0.01
**PtGA>1**			
CDAI vs. DAS28-CRP	-3.19	-3.71,-2.67	<0.01
CDAI vs. DAS28-ESR	-3.08	-3.70; -2.47	<0.01
CDAI vs. SDAI	0.03	-0.33; 0.41	0.85
DAS28-CRP vs. SDAI	3,23	2,71; 3,75	<0,01
DAS28-ESR vs. SDAI	3,12	2,49; 3,75	<0,01
**PhGA >1**			
CDAI vs. DAS28-CRP	-2.41	-2.88; -1.94	<0.01
CDAI vs. DAS28-ESR	-1.93	-2.49;1.37	<0.01
CDAI vs. SDAI	-0.01	-0.30;0.27	0.93
DAS28-CRP vs. SDAI	2,39	1,90; 2,89	<0,01
DAS28-ESR vs. SDAI	1,92	1,35; 2,48	<0,01
**GH > 1**			
CDAI vs. DAS28-CRP	-1.70	-2.21; -1.18	<0.01
CDAI vs. DAS28-ESR	-1.36	-1.97; -0.74	<0.01
CDAI vs. SDAI	0.06	-0.33; 0.46	0.75
DAS28-CRP vs. SDAI	1,76	1,23; 2,3	<0,01
DAS28-ESR vs. SDAI	1,42	7,81; 2,07	<0,01

DAS28-CRP: Disease Activity Score 28- C reactive protein; DAS28-ESR: Disease Activity Score 28 -erythrocyte sedimentation rate; SDAI: Simplified Activity Index; CDAI: Clinical Activity Index; TJC: tender joint count; SJC: swollen joint count; CRP: C reactive protein; GH: global health assessment; PtGA: patient’s global assessment; PhGA: physician’s global assessment

The proportion of patients with swollen and painful joint counts out of target is higher for DAS28-ESR and DAS28-CRP when compared to SDAI and CDAI. While the former two have 3% to 10% of patients with > 1 joint involvement, the latter two have only 1%.

Patient assessment (GH and PtGA) was elevated in all indices, including SDAI and CDAI. But, in the latter two, the rate was lower than DAS28-ESR and DAS28-CRP. While DAS28-CRP and DAS28-ESR showed patient pain assessment rates between 40 to 50%, SDAI and CDAI showed around 14% (14,4% and 14,7%, respectively). Although not part of the Boolean method, physician assessment showed higher rates in DAS28 and less residual activity in SDAI and CDAI (10%). SDAI and CDAI had a lower proportion of patients who did not fulfill the Boolean remission criteria in all components (Table 5).

Finally, the estimated difference rates between each index showed statistical significance between DAS28-CRP and DAS28-ESR when compared to CDAI and SDAI (*p*<0.01), confirming a higher residual activity by DAS28, especially DAS28-CRP. No statistical difference was observed between CDAI and SDAI (Table 6).

When considering the absolute values of components, the minimum and maximum were described in Table 7.

**Table 7:** Minimum and maximum values of components at remission and low activity.

**Remission–Components**	**DAS28-ESR (n = 243)**	**DAS28-CRP (N = 353)**	**SDAI (n = 180)**	**CDAI (n = 223)**
**TJC**	0–8	0–8	0–3	0–2
**SJC**	0–8	0–8	0–2	0–2
**CRP**	NA	0.01–8.7	0–3.0	NA
**ESR**	1–41	NA	NA	NA
**GH**	0–92	0–92	NA	NA
**PtGA**	NA	NA	0–3	0–2.7
**PhGA**	NA	NA	0–2	0–2
**SCORE**	0–2.59	0,96–2.59	0–3.3	0–2.8
**Low activity–Components**	**DAS28-ESR (n = 139)**	**DAS28-CRP (n = 147)**	**SDAI (n = 308)**	**CDAI (n = 370)**
**TJC**	0–9	0–8	0–7	0–7
**SJC**	0–20	0–13	0–8	0–8
**CRP**	NA	0.01–25	0–25	NA
**ESR**	2–95	NA	NA	NA
**GH**	0–84	0–100	NA	NA
**PtGA**	NA*	NA	0–9,6	0–10
**PhGA**	NA	NA	0–6	0–6
**SCORE**	2.61–3.18	2.6–3.19	3.35–11	2.8–10

DAS28-CRP: Disease Activity Score 28- C reactive protein; DAS28-ESR: Disease Activity Score 28 -erythrocyte sedimentation rate; SDAI: Simplified Activity Index; CDAI: Clinical Activity Index; TJC: tender joint count; SJC: swollen joint count; CRP: C reactive protein; GH: global health assessment; PtGA: patient’s global assessment; PhGA: physician’s global assessment.

*NA: not applicable

Although DAS28-CRP had 90% (319) and 96.8% (342) of the patients with up to 1 swollen joint and 1 tender joint, respectively, 10% (34) had up to 8 swollen joints in remission. CRP ≤ 1.0 mg/dL was found in 80.4% (289) of the patients, and almost one-fifth showed values greater than 1. Considering DAS28-ESR, 94% (229) of the patients had up to 1 tender joint, and 93.4% (227) had up to 1 swollen joint. Nevertheless, approximately 6% had up to 8 both swollen and tender joints.

When considering SDAI and CDAI, 98.8% (178) and 98.6% (220) of the patients presented up to 1 swollen joint, respectively. The maximum tender joint count was 3 for SDAI and 2 for CDAI. CRP was ≤ 1 mg /dL in 85.5% (154) of patients evaluated by SDAI. CRP > 1mg/dL was observed in similar proportions in SDAI and DAS28-CRP (14,5% and 19,6% of patients, respectively). CRP was the only component that has shown more residual activity in CDAI compared to DAS28, but it is important to note that CRP is not part of the CDAI.

When considering the low activity, a more significant number of affected joints was observed by DAS28-ESR, pointing up to 20 swollen joints and 13 by DAS28-CRP, while SDAI and CDAI showed a maximum of 8 swollen joints. But it is also important to note there were no newer validated criteria for low activity other than the indices themselves (Table 7).

**Fig 2:**
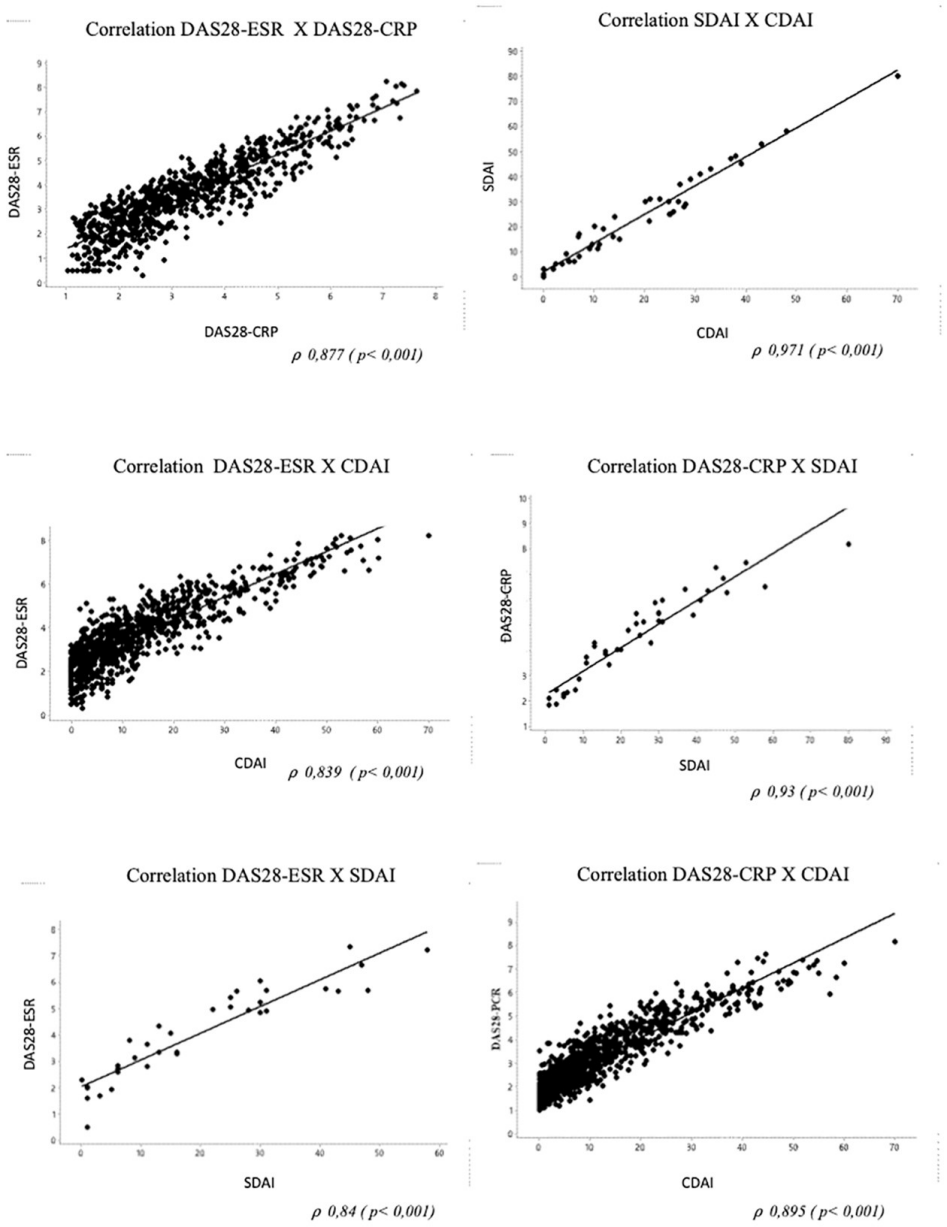
Correlation among the composite disease activity indices.

Correlation among the indices was shown in Fig 2, with the strongest correlation between CDAI and SDAI (*⍴* 0.971, p <0.001).

Table 8 shows the agreement between the indices. The best agreement occurred between SDAI and CDAI (*k* = 0.88; CI 95% 0.86–0.90).

**Table 8:** Agreement among disease activity indices.

Agreement between indices according to disease classification	
	Weighted Kappa (CI 95%)
**DAS28 ESR X DAS28 CRP**	0.63 (0.59–0.67)
**DAS28 ESR X SDAI**	0.60 (0.57–0.64)
**DAS28 ESR X CDAI**	0.60 (0.57–0.64)
**DAS28 CRP X SDAI**	0.70 (0.67–0.73)
**DAS28 CRP X CDAI**	0.66 (0.63–0.69)
**SDAI X CDAI**	0.88 (0.86–0.90)

DAS28-CRP: Disease Activity Score 28- C reactive protein; DAS28-ESR: Disease Activity Score 28 -erythrocyte sedimentation rate; SDAI: Simplified Activity Index; CDAI: Clinical Activity Index.

When comparing sensibility and specificity to achieve remission by the Boolean method, CDAI and SDAI presented higher sensitivity, specificity, and accuracy when compared to other indices (Table 9).

**Table 9:** Comparison of sensitivity and specificity among indices.

Remission	Sensibility	CI 95%	Specificity	CI 95%	Accuracy	*p*
**DAS28-ESR**	73%	0.65;0.80	82%	0.80;0.85	81%	<0.001
**DAS28-CRP**	97%	0.93;0,99	72%	0.69;0.75	76%	<0.001
**CDAI**	90%	0.84–0.94	90%	0.88–0.91	90%	<0.001
**SDAI**	92%	0.91–0.96	93%	0.91–0.95	93%	<0.001

DAS28-CRP: Disease Activity Score 28- C reactive protein; DAS28-ESR: Disease Activity Score 28 -erythrocyte sedimentation rate; SDAI: Simplified Activity Index; CDAI: Clinical Activity Index.

## Discussion

This report is the first large Brazilian cohort study with patients with RA, representing almost all regions of the country that describes demographic, clinical, and therapeutics features of patients with RA and allows comparison of disease activity indices in a real-life setting. Although several studies support the importance of composite disease activity indices, there are limited data related to Brazil. This is the first national study with more than one thousand patients that demonstrates the presence of higher residual activity by DAS28-CRP and DAS28-ESR and encourages the use of SDAI and CDAI as indices for daily practice as an alternative to DAS28.

In this study, considering remission, only 117 of the 1116 patients (10.4%) analyzed were concordant for remission by the four indices simultaneously, and 139 (13.4%) were in remission by the Boolean method. These findings differ from other studies, in which remission may be possible in up to 50% of patients with RA with the appropriate treatment strategy [32]. According to Aga et al., remission could be achieved in 75 to 80% of patients treated with T2T and early diagnosis [33]. In the present cohort, around 50% of patients showed remission in at least one index, which is a low percentage compared to previously published studies. Although, it is essential to consider that it is a real-world study not intended to properly apply the T2T strategy, despite the management remaining aligned with the goal of remission or low disease activity. In addition, the patients have established disease with more than ten years of symptoms, worse prognosis factors, including antibody positivity, delay in starting therapy, and several previous treatment regimens that contribute to lower remission rates [23].

Based on the Boolean method of remission, residual activity was present in all indices evaluated but lesser in SDAI and CDAI. The estimated difference rate in each component was superior in DAS28-CRP and DAS28-ESR when compared with CDAI and SDAI (p < 0,05), with no difference between the latter. In this study, up to 8 tender or swollen joints were observed by DAS28 remission status, a much higher value than the threshold for remission by the Boolean method, which is a concerning result once swollen joint count is directly related to structural and irreversible damage progression in the longer term [34].

Therefore, several studies of composite disease activity indices have already suggested overestimated remission by DAS28, especially DAS28-CRP, even considering stricter cut-offs [17, 35]. According to Aletaha et al., even a cut-off value lower than 2.6 can allow up to 12 swollen joints and disease activity [6, 35–38]. CDAI and SDAI are more stringent and reliable than DAS28 remission criteria, and different types of therapies do not influence them [39, 40]. In a recent paper, Schoels et al. analyzed three clinical trials of tocilizumab to describe residual activity by DAS28. The cut-off of DAS28-PCR and DAS28-ESR was reduced to <1,9 and < 2,2, respectively, and residual activity was still detected in DAS28-CRP, mainly based on swollen joints, an important component to predict radiologic progression [6]. This evidence suggests not only problems with the cut-off points or weight of inflammatory markers but with the formula [6].

Interestingly, pain assessment parameters, especially the global health assessment, are elevated in all indices at remission; however, PtGA used in SDAI and CDAI can better classify these patients and show less residual activity. The established disease with irreversible joint damage, comorbidities, and polypharmacy in these patients may contribute to higher pain scores and activity status [26]. On the other hand, PhGA parameters are lower in SDAI and CDAI when compared to DAS28-ESR and DAS28-CRP and demonstrate a significant estimated difference rate. In addition, they can suggest discordance between patient and physician with the first overlapping the second, which can occur in 18 to 49% of cases [29].

Although residual symptoms were not evaluated, it is noteworthy that pain, fatigue, and global health assessment described in Table 4 are present in this population at considerable rates (median 40), consistent with higher disease activity levels found in the study.

This study showed good correlation and concordance among indices, supporting the literature data, which already recognized the positive correlation and agreement among indices, especially between SDAI and CDAI [36, 41–43]. SDAI became part of the remission criteria of ACR/EULAR and an important tool for clinical trials, while CDAI has been more recommended for clinical practice [13]. Since they consider the same variables in the formula, a good correlation was expected, as well as good agreement, especially between SDAI and CDAI (*k*:0,88), which correspond to the linear sum and have the same component weight [36, 41–43].

Finally, SDAI and CDAI were closer to the Boolean remission concept, and both showed lower residual activity when compared with DAS28-ESR and DAS28-CRP. DAS28 and SDAI were performed in approximately 85%. In comparison, CDAI was performed in 99% of patients and presented a maximum of two joints affected in remission, favoring the latter use routinely when laboratory tests are unavailable and considering its performance in almost the totality of patients [36, 41–43]. SDAI and CDAI showed higher sensitivity and specificity for remission based on the Boolean method. CDAI can be more attractive in developing countries with social and economic limitations, especially given its better adherence in a real-life setting [14, 22, 44–48].

The study presents several limitations, including the impossibility of comparison of the indices with the gold standard imaging method, classification of residual activity only by disease activity scores, no control of variables, and no standardization of laboratory tests. However, this real-world study can recognize local challenges and allow rational allocation of resources and the development of strategies that better adjust to the local reality.

## Conclusions

Although all composite disease activity indices can be used in clinical practice and showed good agreement, CDAI and SDAI showed less residual activity, higher sensibility, and specificity for remission based on the Boolean method. In developing countries, the availability of a low-cost and readily accessible index such as CDAI may be better for public health planning strategy. This study encourages using SDAI and CDAI indices for daily practice as an alternative to DAS28.
